# The Involvement of Amino Acid Metabolism in the Mechanisms of Salt Tolerance Adaptation in *Medicago sativa* and *Medicago truncatula*

**DOI:** 10.3390/plants14060929

**Published:** 2025-03-15

**Authors:** Sicong Shen, Ling Pan, Junhao Li, Jing Wang, Irshad Ahmad, Huhu Liu, Yuyu Bai, Bowen Kang, Juncheng Yin, Yang Gao, Yiwen Lu, Xiaoshan Wang

**Affiliations:** College of Animal Science and Technology, Yangzhou University, Yangzhou 225009, China; 15205271258@163.com (S.S.); panling1199@126.com (L.P.); ljhyttx@163.com (J.L.); wang0217j@163.com (J.W.); irshadgadoon737@yahoo.com (I.A.); 15052507537@163.com (H.L.); 15718684401@163.com (Y.B.); kbw20002023@163.com (B.K.); junchengyin@outlook.com (J.Y.); xx_gaoyang@163.com (Y.G.); 18261898683@163.com (Y.L.)

**Keywords:** amino acid metabolism, *Medicago sativa*, *Medicago truncatula*, salt stress, transcriptomics and proteomics

## Abstract

Amino acid metabolism constitutes a major metabolic pathway in plants, playing an important role in the modulation of plant responses to stress. In this study, we investigated the amino acid metabolism responses of *M. sativa* (*Medicago sativa* L.) and *M. truncatula* (*Medicago truncatula* L.) plants under salt stress using transcriptomic and proteomic approaches to elucidate their salt stress tolerance mechanisms in relation to the regulation of amino acid homeostasis. Transcriptome and proteome sequencing followed by Kyoto Gene and Genome Encyclopedia enrichment analysis revealed 34 differentially expressed genes and 45 differentially expressed proteins involved in valine, leucine, and isoleucine degradation, tyrosine metabolism, and glutathione metabolism. Significant differences were observed in the expression of glutathione S-transferase (*GST*) within the glutathione metabolic pathway between *M. sativa* and *M. truncatula*. The induction of valine, leucine, and isoleucine metabolism, aldehyde dehydrogenases (*ALDHs*), and alanine-glyoxylate aminotransferases (*AGXTs*), involved in intracellular reactive oxygen species scavenging, also significantly differed under salt stress. Significant differences were identified in the expression of tyrosine decarboxylases (*TDCs*) involved in tyrosine metabolism, which are responsible for tyramine biosynthesis and can enhance plant tolerance to salt stress. This study delved into the effects of amino acid metabolism on the salt tolerance mechanisms of *M. sativa* and *M. truncatula*, which is crucial in guiding the future breeding of salt-tolerant alfalfa varieties.

## 1. Introduction

Salt stress is a major abiotic stress that adversely affects crop growth and yield worldwide [1]. Based on the available data, salt stress affected nearly 30,000 ha of agricultural land in 2023, impacting agricultural output and agroecosystem sustainability [2,3,4,5]. Therefore, it is imperative to investigate the plant salt tolerance mechanism and examine the metabolic pathways associated with salt stress adaptation to enhance salt tolerance.

Amino acid metabolism is an essential component of numerous downstream metabolic processes in plants. Plant adaptation to salt stress is regulated by numerous metabolic pathways, with the regulation of amino acid metabolism being a crucial component. Previous research has shown that salt stress changes the expression of key genes for amino acid metabolism and increases the biosynthesis of amino acids [6]. In *Sapium sebiferum* (*Triadica sebifera*), differentially expressed proteins (DEPs) under salt stress primarily participated in amino acid metabolic pathways, and amino acids and other soluble molecules primarily accumulated in the leaves of salt-tolerant *S. sebiferum*, resulting in enhanced salt tolerance [7]. Researchers found a significant up-regulation of amino acid metabolism-related genes in salt-tolerant varieties of wild soybean (*Glycine soja*) under salt stress [8].

Amino acids, key structural and metabolic compounds in plants, are indispensable in plant salt tolerance [9,10,11]. Free amino acids, such as glutamate (Glu), rapidly accumulate in plants under salt stress [12,13]. Previous studies have shown that Glu is central to amino acid metabolism and is one of the important substrates for the biosynthesis of other amino acids [14]. Wang et al. investigated the mechanisms of salt tolerance in the non-secretory mangrove *Kandelia candel*. They found that glutamate contents significantly increased under salt stress, acting as an osmotic regulator to protect the plant from salt toxicity [15]. In salt-tolerant varieties of tomato (*Solanum lycopersicum*), Cano et al. found that the contents of valine, leucine, and isoleucine increased significantly with increasing concentrations of salt [16]. Lu et al. found that a tyrosine-related tyrosine-specific phosphatase, *PdPTP1*, was significantly up-regulated in *Prunus populiflora* under salt stress, thus enhancing the tolerance of the plant to salt by affecting the balance between ions and reactive oxygen species (ROS) [17]. Amino acids also serve as precursors for the biosynthesis of many signaling molecules that enhance salt tolerance in different plants [18]. For example, TOR signaling in plants is activated by 15 amino acids and regulated by a single amino acid (leucine) [19,20]. Leucine, isoleucine, and tyrosine exhibit strong metabolic and functional associations and, thus, function together in biological signaling under salt stress [21,22,23].

Exogenous application of amino acids has also been shown to alleviate salt stress in plants. Exogenous proline, a key indicator of salt stress severity and tolerance in plants, provides protection by reducing cellular damage from salt stress [24,25]. In addition, the exogenous application of glycine (Gly) and Glu can increase the content of Ca^2+^ in plants and reduce the toxicity of Na^+^ ions, which results in improved tolerance to salt [26]. Yao et al. demonstrated that the exogenous applications of Cys, Ser, and Met activate Ca^2+^ channels to mitigate the adverse effect of salt stress in various crops [27]. Abdelkader et al. exogenously applied six amino acids, with Gly, methionine (Met), and Pro, significantly increasing the activity of peroxidase (POD), balancing the ionic state, and promoting the biosynthesis of chlorophyll *a* and chlorophyll *b*, which, in turn, improved plant growth and development under salt stress [28].

*M. sativa* (*Medicago sativa* L.) is a leguminous perennial crop cultivated on more than 6 million ha [29]. It is considered one of the world’s most economically important crops for agriculture and animal husbandry [30,31]. *M. truncatula* (*Medicago truncatula* L.) is a model legume widely studied in recent years; it features a small genome, self-pollination, a short life cycle, a high fruiting rate, and a stable genetic transformation system [32]. Salt stress is among the most important challenges affecting the cultivation of *M. sativa* and *M. truncatula*. Previous research demonstrated that *M. sativa* and *M. truncatula* are highly sensitive to salt during seed germination and seedling growth [33]. However, the salt tolerance mechanisms in the seedlings are very complex and still poorly understood [34]. Therefore, understanding *M. sativa* and *M. truncatula* responses to salt stress and identifying salt tolerance genes are crucial for their subsequent breeding and improvement towards increased salt stress tolerance.

In this study, we combined transcriptomic and proteomic analyses to integratively investigate and analyze for the first time the effects of amino acid metabolic pathways on seed germination and seedling growth of *M. sativa* and *M. truncatula* under salt stress. Our findings aim to establish a framework for genes associated with salt stress resistance in *M. sativa* and *M. truncatula*, serving as a foundation for the subsequent breeding of salt-tolerant cultivars of *M. sativa* and *M. truncatula* to enhance productivity in saline soils.

## 2. Results

### 2.1. Effects of NaCl Stress on the Germination of M. sativa (Medicago sativa L.) and M. truncatula (Medicago truncatula L.) Seeds

**Figure 1:**
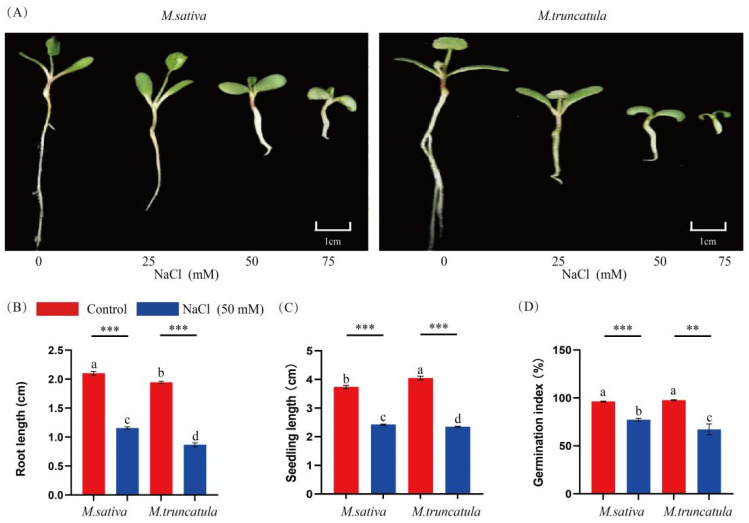
Effects of different NaCl concentrations after 7 days of treatment on *M. sativa* (*Medicago sativa* L.) and *M. truncatula* (*Medicago truncatula* L.) seedling morphology (**A**). Effects of 50 mM NaCl after 7 days of treatment on root length (**B**), seedling length (**C**), and germination index (**D**). Different letters indicate significant differences between species/treatment groups. (*p* < 0.05). In the *t*-test, ** *p* < 0.01, *** *p* < 0.001.

Both *M. sativa* and *M. truncatula* are leguminous plants but have different adaptative capacities to salt stress. To assess their ability to adapt to NaCl stress, we conducted germination experiments with *M. sativa* and *M. truncatula* seeds under varying NaCl concentrations. The treatment of both *M. sativa* and *M. truncatula* with NaCl significantly inhibited seedling growth 7 days after seed germination. *M. truncatula* was more sensitive to NaCl stress compared to *M. sativa*, which demonstrated higher tolerance during seed germination (Figure 1A). Compared with the control, the root length, seedling length, and germination index of *M. sativa* seedlings were reduced by 44.97%, 35.12%, and 25.5%, respectively. On the other hand, the root length, seedling length, and germination index of the *M. truncatula* seedlings were reduced to a greater extent compared to *M. sativa*, by 55.43%, 42.03%, and 41.32%, respectively (Table S1; Figure 1B–D).

**Figure 2:**
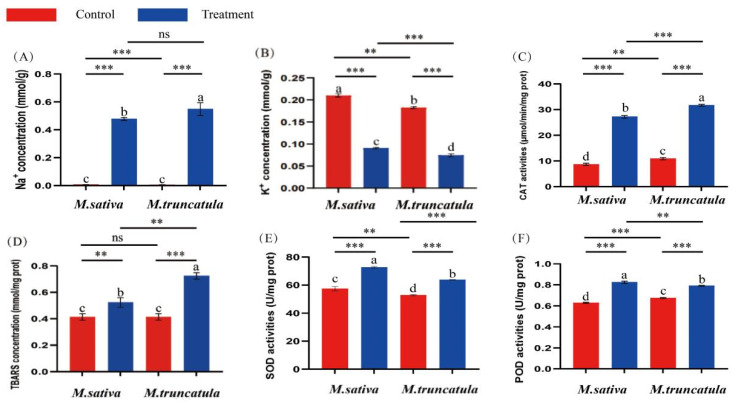
Biochemical indices of *M. sativa* and *M. truncatula* seedlings treated with 50 mM NaCl for 7 days. Na^+^ content (**A**), K^+^ content (**B**), CAT activity (**C**), TBARS content (**D**), SOD activity (**E**), and POD activity (**F**). The units of measurement are expressed as mmol/g, where ‘g’ denotes the fresh weight of the sample. Different letters indicate significant differences between the species/treatment groups (*p* < 0.05). In the *t*-test, ** *p* < 0.01, *** *p* < 0.001, ns *p* > 0.05. CAT, catalase; TBARS, tiobarbituric acid-reactive substances; POD, peroxidase; SOD, superoxide dismutase.

Under NaCl stress conditions, excess Na^+^ in the plant cells damages the cell membrane system, which results in the production of TBARS. Furthermore, NaCl stress induces oxidative stress in plants and alters the activities of antioxidant enzymes. In this study, treatment with 50 mM NaCl significantly increased Na^+^ while reducing K^+^ contents in *M. sativa* and *M. truncatula* seedlings. The Na^+^ content of *M. truncatula* seedlings treated with 50 mM NaCl for 7 days was significantly higher (*p* < 0.05) than that of the *M. sativa* seedlings (Figure 2A). In contrast, *M. sativa* seedlings treated with 50 mM NaCl for 7 days had significantly higher K^+^ content (*p* < 0.05) than *M. truncatula* seedlings (Figure 2B). Simultaneously, the content of TBARS, as well as the activities of CAT, SOD, and POD, were significantly higher in the *M. sativa* and *M. truncatula* seedlings treated with 50 mM NaCl for 7 days (Figure 2C–F). Compared with the *M. sativa* seedlings, *M. truncatula* had a significantly higher TBARS content and CAT activity (*p* < 0.05) (Figure 2C,D). In contrast, it had a reduction in the activities of SOD and POD (Figure 2E,F). These results further elucidated the physiological mechanisms underlying the differential responses of *M. sativa* and *M. truncatula* to salt stress.

### 2.2. Effects of NaCl Stress on Amino Acid Metabolism in M. sativa and M. truncatula Seedlings

Plants respond to salt stress by altering the biosynthesis of amino acids at the gene expression level to maintain growth and metabolism and adapt to the external environment. In this study, a transcriptome sequencing analysis was performed on the *M. sativa* and *M. truncatula* seedlings treated with 50 mM NaCl to investigate the changes in amino acid metabolic pathways and the expression of key genes under NaCl stress. In total, 75.76 GB of clean reads data were obtained by sequencing, revealing that the data from the NaCl-treated *M. sativa* and *M. truncatula* seedlings were highly correlated with those of the control (R^2^ > 0.8). This indicated the reliability of the biological replicates (Figure S1). The principal component analysis (PCA) primarily dispersed the distribution of *M. truncatula* seedlings treated with 50 mM NaCl compared to the control along the PC2 axis (Figure S2), indicating significant differences in their transcriptomes under 50 mM NaCl.

When analyzing the data, genes with a *p* value < 0.05 and an absolute change in expression ≥2-fold between the treatments were considered significant DEGs. A KEGG pathway heatmap was drawn based on the expression of DEGs in the *M. sativa* and *M. truncatula* seedlings after treatment with 50 mM NaCl (Figure S3). Each block represents the expression value of its gene. Red represents up-regulated expression, and green represents down-regulated expression. A total of 14 DEGs were involved in amino acid metabolism. They primarily included DEGS involved in leucine and isoleucine metabolism and tyrosine metabolism, among others, and 20 DEGs in the metabolism of other amino acids. This primarily included glutathione metabolism, among others. Notably, the 50 mM NaCl treatment significantly decreased the expression of DEGs involved in amino acid metabolism in *M. sativa* and *M. truncatula* compared to the control. In contrast, the expression of differentially expressed genes (DEGs) related to the metabolism of other amino acid types did not exhibit significant changes.

The DEGs involved in the amino acid metabolism KEGG pathways were analyzed statistically (Table S2). No DEGs involved in amino acid metabolism were identified in *M. sativa* under 50 mM NaCl (S0 vs. S50). Still, a total of 11 DEGs were found to be significantly expressed in *M. truncatula* (T0 vs. T50). Two of these DEGs, including aldehyde dehydrogenase family 7 member A1 (*ALDH7A1*) and aldehyde dehydrogenase family 3 member H1 (*ALDH3H1*), were up-regulated. Nine DEGs were down-regulated, including alanine-glyoxalate aminotransferase 2 homologue 3, mitochondrial (*AGXT 2 3*), alanine-glyoxalate aminotransferase 2 homologue 1, mitochondrial (*AGXT 2 1*), aldehyde dehydrogenase family 2 member B4 (*ALDH2B4)*, aldehyde dehydrogenase family 2 member B4, mitochondrial isoform X1 (*ALDH2B4 X1*), methylcrotonyl-CoA carboxylase subunit alpha, mitochondria (*MCC*), 2-isovaleric acid dehydrogenase subunit alpha 1, mitochondria (*2-ODH*), isovaleryl-CoA dehydrogenase, mitochondria (*IVD*), acetyl-CoA acetyltransferase, cytoplasm 1 (*ACAT 1*), benzaldehyde dehydrogenase, and mitochondrial (*BALDH*). A comparison of the *M. sativa* and *M. truncatula* controls (S0 vs. T0) identified a total of three DEGs involved in tyrosine metabolism. One DEG, which encoded alcohol dehydrogenase 1 (*ADH 1*), was up-regulated. In contrast, two DEGs were down-regulated: those that encoded tyrosine decarboxylase 1 (*TDC 1*) and tyrosine decarboxylase 2 (*TDC 2*). Three amino acid metabolic pathways, namely degradation of tryptophan, leucine, and isoleucine, tyrosine metabolism, and glutathione metabolism, were identified after the comparison of *M. sativa* and *M. truncatula* seedlings (S50 vs. T50) treated with 50 mM NaCl. Ten DEGs that were involved in tyrosine metabolism and *ADH1*, bifunctional aspartate aminotransferase glutamate/aspartate-prephenate aminotransferase (*PAT*), and alcohol dehydrogenase-like 2 (*ADH 2*) were up-regulated, whereas seven DEGs, including *TDC 1*, *TDC 2,* histidinol-phosphate aminotransferase, chloroplastic (*HPAT*), glutathione S-transferase zeta class (*GST*), aspartate aminotransferase, mitochondrial (*AST*), alcohol dehydrogenase class-3 (*ADH 3*), and 8-hydroxygeraniol oxidoreductase (*8-HGO*), were down-regulated. A total of 13 identified DEGs were involved in glutathione metabolism, including ten DEGs that were up-regulated, encoding for glutathione S-transferase (*GST*), glutathione S-transferase U17 (*GST U17*), glutathione S-transferase L3 (*GST L3*), glutathione S-transferase T1 (*GST T1),* glutathione S-transferase F9 (*GST F9*), glucose-6-phosphate 1-dehydrogenase, cytoplasmic isoform (*G6PD*), inactive glucose-6-phosphate 1-dehydrogenase 4, chloroplastic (*G6PD4*), glucose-6-phosphate 1-dehydrogenase 5, cytoplasmic (*G6PD5*), glutathione S- transferase *DHAR3*, chloroplastic (*DHAR3*), and leucine aminopeptidase 1 (*LAP 1*). A total of three DEGs, including glutathione S-transferase U22 (*GST U22*), glutathione S-transferase U40 (*GST U40*), and glutathione S-transferase U9 (*GST U9*), were down-regulated. Seven DEGs were involved in the degradation of valine, leucine, and isoleucine, with two, *ALDH7A1* and *ALDH3H1*, being up-regulated and five, including *AGXT 2 3*, *AGXT 2 1*, *ALDH2B4*, *ALDH2B4 X1*, and *ACAT 1*, being down-regulated. Specific information on the DEGs identified to be involved in amino acid metabolism is shown in Table S3. These results primarily revealed *GSTs* as the DEGs involved in glutathione metabolism, with five up-regulated and four down-regulated. The DEGs involved in tyrosine metabolism primarily encoded *TDCs* and were down-regulated. The DEGs responsible for valine, leucine, and isoleucine degradation primarily encoded *ALDHs*, with two up-regulated and two down-regulated. In summary, the *GSTs*, *TDCs*, and *ALDHs* may be the key DEGs that affect salt tolerance in *M. sativa* and *M. truncatula*.

**Figure 3:**
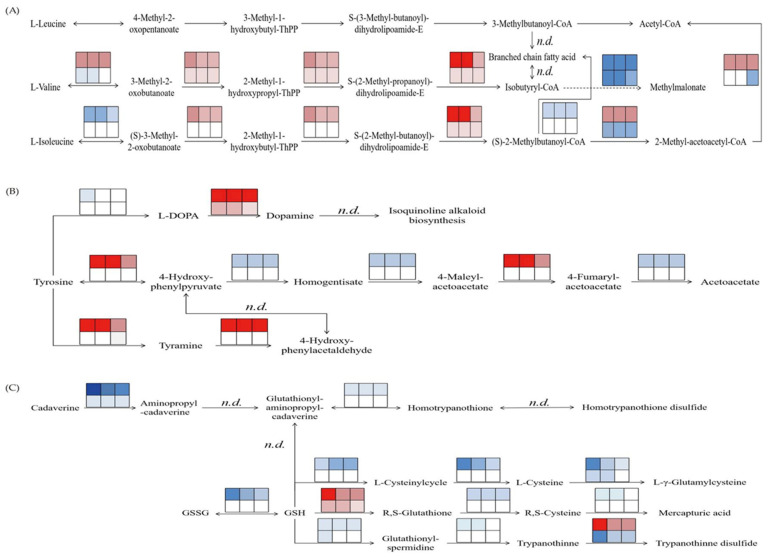
Expression map of the significantly enriched amino acid pathways. (**A**) Metabolism of leucine and isoleucine in *M. sativa* (*Medicago sativa* L.). (**B**) Metabolism of tyrosine in *M. truncatula* (*Medicago truncatula* L.). (**C**) Metabolism of glutathione in *M. truncatula*. The top three blocks in each sub-table in the figure represent *M.sativa* or *M. truncatula* treated with 50 mM NaCl, and the bottom three represent the control with three replicates. Squares, genes involved in the pathway; red, up-regulation; blue, down-regulation.

We performed GSEA analyses to investigate the significance of the amino acid metabolic pathways in the transcriptome of salt-stressed *M. sativa* and *M. truncatula*. As a result, a total of two amino acid metabolism-associated KEGG pathways were found to be significantly differentially expressed, namely, the valine, leucine, and isoleucine degradation pathways identified in the T0 vs. T50 comparison (NES = 1.887 and FDR = 0.045, *p* = 0.000), and the tyrosine metabolism pathway identified in the S50 vs. T50 comparison (NES = 1.769 and FDR= 0.085, *p* = 0.043) (Figure S4). A flow chart of the significantly differentially expressed amino acid metabolic pathways is shown in Figure 3. The pathway map reveals that the DEGs involved in leucine, isoleucine, and valine metabolic pathways were primarily clustered downstream of the pathways, and their levels of expression tended to initially increase and then decrease. The DEGs involved in tyrosine and glutathione metabolism were primarily clustered upstream of the pathway and showed a trend of decreasing expression.

To investigate in greater detail the role of amino acid metabolism in salt-stressed *M. sativa* and *M. truncatula* seedlings, proteome sequencing was performed on controls and seedlings treated with 50 mM NaCl for 7 days. As a result, a total of 266,819 spectra were obtained, including 71,265 matched spectra, 42,350 peptides, 7192 identified proteins, and 7154 quantifiable proteins (Table S4). The principal component analysis (PCA) results showed that *M. truncatula* treated with 50 mM NaCl for 7 days clustered predominantly along the PC2 axis compared to the control. Conversely, the *M. sativa* and *M. truncatula* controls were primarily distributed along the PC1 axis (Figure S5). This indicated a difference in the expression of proteins between *M. sativa* and *M. truncatula* under salt stress conditions.

When analyzing the data, genes with a *p* value < 0.05 and an absolute change in expression ≥ 2-fold were considered significant DEPs. To identify the proteins involved in amino acid metabolism, a KEGG heatmap based on proteomics data was drawn according to the expression results of the DEPs (Figure S6). Each plot represents the expression value of a protein, with red representing up-regulated expression and blue representing down-regulated expression. Based on the results, the DEPs were primarily involved in amino acid metabolism, including leucine, isoleucine, tyrosine, and the metabolism of other types of amino acids. It primarily includes glutathione metabolism among others. Compared with *M. truncatula*, the DEPs in *M. sativa* showed a significant upward trend in expression. Under the 50 mM NaCl treatment, the expression of DEPs involved in amino acid metabolism in *M. sativa* and *M. truncatula* significantly decreased compared with the control. In contrast, the expression of most DEPs involved in the metabolism of other amino acids did not change significantly (Figure S6).

No DEPs involved in amino acid metabolism were identified in the *M. sativa* seedlings treated with 50 mM NaCl in comparison to the control (S0 vs. S50) in the proteomic analyses. As shown in Table S5, only one DEP (*ALDH7A1*) involved in amino acid metabolism was detected in the 50 mM NaCl-treated *M. truncatula* seedlings (T0 vs. T50), which was down-regulated. A comparison between the *M. sativa* and *M. truncatula* control seedlings (S0 vs. T0) revealed a total of 21 DEPs involved in glutathione metabolism. Of these, 14 were up-regulated (*LAP 1*, *GST*, *GST U17*, glutathione S-transferase *DHAR1*, mitochondrial (*DHAR1*), ascorbate peroxidase isoform 2 (*APX 2*), glutathione reductase, cytosolic (*GR*), thylakoid lumenal 29 kDa protein, chloroplastic (*TL 29*), probable glutathione S-transferase parC (*PARC*), *GST L3*, *GST F9*, glutathione hydrolase 1 (*GGT1*), GST 3, homoglutathione synthetase (*GSH*), glucose-6-phosphate 1-dehydrogenase, cytosolic phosphate 1-dehydrogenase, cytoplasmic isoform (*G6PD*)), while 7 were down-regulated (*GST U9*, glutathione S-transferase U22 (*GST U22*), glutathione S-transferase U42 (*GST U42*), glutathione reductase, chloroplastic/mitochondrial (*GR*), 6-phosphogluconate dehydrogenase, decarboxylating 1, and chloroplastic (*6PGDH1*)). In the comparison between *M. sativa* and *M. truncatula* seedlings treated with 50 mM NaCl (S50 vs. T50), three KEGG amino acid metabolism pathways were identified, particularly tryptophan metabolism, with a total of three up-regulated DEPs and eight down-regulated DEPs present. Tyrosine metabolism (including one up-regulated DEP and eight down-regulated DEPs) and glutathione metabolism (including a total of 16 up-regulated DEPs and nine down-regulated DEPs) were also overrepresented. The specific information on the DEPs involved in amino acid metabolism is shown in Table S6. The results revealed the involvement of 12 *GSTs* in glutathione metabolism, with six exhibiting up-regulation and six down-regulation. The *TDCs* were primarily involved in tyrosine metabolism and were down-regulated. *ALDHs* were the DEPs that were primarily responsible for the degradation of tryptophan, leucine, and isoleucine., with three up-regulated and two down-regulated. The results of the proteomic analysis were consistent with those of the transcriptomic analysis. Therefore, the *GSTs*, *TDCs*, and *ALDHs* may play an indispensable role in the metabolism of DEPs, thus affecting the mechanisms of salt tolerance in *M. sativa* and *M. truncatula*.

This study further investigated the role of amino acid metabolism in the *M. sativa* and *M. truncatula* seedlings treated with 50 mM NaCl, using combined transcriptomic and proteomic analyses. Differential expression analyses at the gene and protein levels identified a total of 563 DEGs/DEPs. Moreover, 19 KEGG pathways were significantly differentially expressed as determined by KEGG enrichment analyses. The identified KEGG pathways were categorized and summarized, and it was found that the highest number of DEGs/DEPs were involved in amino acid metabolism, including amino acid metabolism (This primarily included leucine and isoleucine metabolism and tyrosine metabolism) and other amino acid metabolism(This primarily included glutathione metabolism). Among them, two DEGs/DEPs were involved in amino acid metabolism, and 11 DEGs/DEPs were involved in the metabolism of other amino acids (Table S7). The combined transcriptomic and proteomic analyses unequivocally revealed that the metabolism of amino acids played an important role in *M. sativa* and *M. truncatula* under salt stress.

### 2.3. Quantitative Analysis of Amino Acid Concentrations

**Figure 4:**
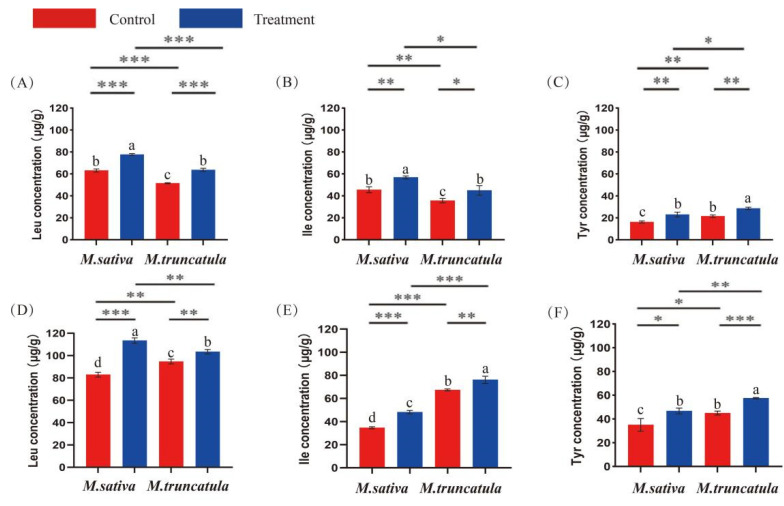
Determination of tyrosine, leucine, and isoleucine contents in the roots (**A**–**C**) and leaves (**D**–**F**) of *M. sativa* (*Medicago sativa* L.)and *M. truncatula (Medicago truncatula* L.) seedlings. (**A**) Leu content in the roots, (**B**) Ile content in the roots, (**C**) Tyr content in the roots, (**D**) Leu content in the leaves, (**E**) Ile content in the leaves, (**F**) Tyr content in the leaves. The units of measurement are expressed as μg/g, where ‘g’ represents the fresh weight of the sample. Different letters indicate significant differences between treatment groups. In the *t*-test, * *p* < 0.05, ** *p* < 0.01, *** *p* < 0.001.

Based on the transcriptome and proteome analyses results, the contents of three amino acids—Tyr, Leu, and Ile—were determined in the leaves and roots of *M. sativa* and *M. truncatula* seedlings grown under control conditions of 50 mM NaCl for 7 days. As shown in Figure 4, Leu was the most abundant amino acid in *M. sativa* and *M. truncatula*, followed by Ile and Tyr. Compared to the control, the contents of all three amino acids in the leaves and roots of the *M. sativa* and *M. truncatula* seedlings were significantly (*p* < 0.05) increased under the 50 mM NaCl treatment. Interestingly, the content of Leu in the roots of seedlings was significantly higher than that in the *M. truncatula* seedlings. In contrast, *M. sativa* Tyr content was significantly lower compared to both the leaves and roots of *M. truncatula* seedlings (*p* < 0.05). This suggests that Leu and Tyr may be important amino acids that affect the adaptation and tolerance of *M. sativa* and *M. truncatula* to salt stress, respectively.

### 2.4. Exogenous Application of Leu, Ile, and Tyr

The three amino acids were exogenously applied to the plants to verify the effects of Leu, Ile, and Tyr on potentially enhancing the salt tolerance of *M. sativa* and *M. truncatula*. As shown in Table 1, Table 2 and Table 3, *M. sativa* and *M. truncatula* root and whole seedling lengths increased to different degrees (*p* < 0.05) after the application of the three amino acids. As compared with the control, the content of TBARS and the activities of SOD, POD, and CAT were significantly reduced (*p* < 0.05), which indicated that the exogenous application of Leu, Ile, and Tyr significantly improved the growth and development of the *M. sativa* and *M. truncatula* seedlings. Exogenous Leu, Ile, and Tyr application significantly increased root and seedling lengths in *M. sativa* and *M. truncatula* seedlings compared to 50 mM NaCl treatment for 7 days (*p* < 0.05) while reducing TBARS content and SOD, POD, and CAT activities (*p* < 0.05). This suggests that the exogenous Leu, Ile, and Tyr application can significantly improve salt tolerance in *M. sativa* and *M. truncatula*. A comparative analysis of the effects of the three amino acids on the *M. sativa* seedlings treated with 50 mM NaCl for 7 days showed that the longest root length and seedling length and the lowest activities of SOD, CAT, and POD were observed after the exogenous application of Leu. Interestingly, the *M. truncatula* seedlings exhibited the longest root length and seedling length and the lowest activities of SOD and CAT and content of TBARS after the exogenous application of Tyr. In conclusion, the exogenous application of Leu, Ile, and Tyr significantly improved *M. sativa* and *M. truncatula* salt tolerance. Among them, Leu had the most significant effect on *M. sativa*. In contrast, Tyr had the most significant impact on *M. truncatula*, which suggests that Leu and Tyr may be key constituents that affect the salt tolerance of *M. sativa* and *M. truncatula*.

## 3. Discussion

### 3.1. Changes in the Levels of Reactive Oxygen Species (ROS) in M. sativa (Medicago sativa L.) and M. truncatula (Medicago truncatula L.) Seedlings Under Salt Stress

Reactive oxygen species (ROS) are a class of reactive molecules derived from oxygen that are more reactive than oxygen molecules (O_2_) [35] and play a key role in plant responses to stress and in normal growth and development. In plants, subcellular basal metabolism is the main source of ROS production; this includes aerobic respiration in mitochondria, photosynthesis in chloroplasts, respiration in peroxisomes, and NADPH oxidase activity [36]. SOD and POD enzymes, which are oxidoreductases, are present in plant cells and can scavenge excess O_2_^−^ ions under salt stress, thus protecting the major components of plant cells from oxidative damage. Research has demonstrated that ROS abundance increases in response to biotic and abiotic stresses, leading to an increase in the activities of antioxidant enzymes [37]. In this study, the activities of CAT, SOD, and POD increased significantly in *M. sativa* and *M. truncatula* after treatment with 50 mM NaCl, which suggests an increased abundance of ROS under salt stress.

In plants, TBARS, K^+^, and Na^+^ contents are also markers of intracellular ROS abundance. The main components of TBARS are unsaturated fatty acids, and they are one of the indicators of the degree of damage to the plant cell membrane [38]. The TBARS content of *M. sativa* and *M. truncatula* under salt stress increased significantly in this study when compared with the control. Liu et al. [39] found that in the model plant *Arabidopsis thaliana,* the TBARS content and SOD activity increased under NaCl stress. Yang et al. [40] demonstrated that the SOD activity, MDA content, and photosynthetic rate of wheat (*Triticum aestivum*) increased under salt stress where the increase in SOD activity and photosynthetic rate was greater than that of the MDA content, in agreement with the results of this study. CAT can degrade H_2_O_2_, a form of ROS [41]. Long et al. [42] found that in *M. sativa* and *M. truncatula* under salt stress, the contents of H_2_O_2_, Pro, and abscisic acid (ABA) increased, while the activities of SOD and POD were increased, consistent with the results of this study.

Excessive ROS accumulation can cause damage to plant cells. Consequently, it is essential for plants to preserve cellular ROS homeostasis. This study revealed a significant differential expression of the *GST* genes involved in glutathione metabolism, which contributes to cellular adaptation, enhances cellular antioxidant capacity and reduces the damage by ROS [43]. Tyrosine goes through a nitration reaction associated with ROS signaling in plants, thus reducing the accumulation of ROS [44]. The oligopeptides *LPK* and *LHK* contain other amino acids, such as leucine, which significantly reduce ROS contents [45]. Therefore, by eliminating excess ROS from the cells, amino acid metabolism can mitigate the damage that salt stress causes to *M. sativa* and *M. truncatula* during seed germination.

### 3.2. Relationship Between the Amino Acid Metabolism and Salt Tolerance Mechanisms in M. sativa and M. truncatula

In this study, *M. sativa* and *M. truncatula* seedlings were assessed under salt stress using transcriptomic and proteomic approaches, and the pathways significantly up- or down-regulated in expression were screened through KEGG enrichment analysis. The results revealed significant differences in the expression of amino acid metabolism pathways. Therefore, it was hypothesized that they would play a critical role in the salt tolerance mechanisms of *M. sativa* and *M. truncatula*. Specifically, three amino acid pathways were significantly differentially expressed in the *M. sativa* and *M. truncatula* seedlings under salt stress: glutathione metabolism, valine, leucine, and isoleucine metabolism, and tyrosine metabolism. Marked differential expression of alanine and glutamate metabolism has been observed in *Leymus chinensis* subjected to salt stress. [46]. Bao et al. studied *Suaeda* plants that exhibit innate salt tolerance through metabolomics. They found that the concentration of several amino acids, including Leu and Tyr, increased under salt stress [47]. Simultaneously, tyrosine metabolism and valine, leucine, and isoleucine metabolism were significantly differentially induced [48], which is consistent with the results of this study.

The proteome and transcriptome sequencing results indicated that the proteins and genes involved in glutathione metabolism were primarily *GSTs*. These enzymes, found in plants and animals, interact with *GSH* to catalyze detoxification reactions in plants under a variety of stress conditions, including salt stress, and are involved in the repair of cellular membrane damage and regulating cellular homeostasis, improving salt tolerance. Hernández et al. [49] found a significant differential expression of *GR* and *GSTs* in the glutathione metabolism pathway in *Arabidopsis thaliana* seedlings under salt stress, which is in agreement with the results of this study. The primary genes and corresponding proteins involved in the metabolic pathway for the degradation of tryptophan, leucine, and isoleucine are *ALDHs* and *AGXTs*. *ALDH* is an aldehyde dehydrogenase that primarily catalyzes the conversion of acetaldehyde to acetic acid and is involved in the regulation of osmotic homeostasis and detoxification reactions in plant cells. *AGXT* is an enzyme that primarily converts harmful acetaldehyde to glycine. It is involved in the regulation of peroxisome function and is essential for removing excess ROS from plant cells. The metabolism of valine, leucine, and isoleucine has been suggested to enhance the salt tolerance capacity of *M. sativa* and *M. truncatula*. *TDC* and *ADH* are the primary genes and proteins involved in tyrosine metabolism. *TDC* is a decarboxylase enzyme that can catalyze Tyr’s decarboxylation reaction to produce tyramine, a biologically active compound that plays an important role in plant response to salt stress. *ADH* is a key plant enzyme that catalyzes the reversible reaction between alcohols and aldehydes, thereby reducing the cellular damage caused by alcohols. *ADH* is also a zinc-containing metalloenzyme, with zinc being an essential micronutrient in plant growth that enhances photosynthesis and augments plant stress resistance. Interestingly, the GSEA analysis in this study showed that two amino acid metabolic pathways, valine, leucine, and isoleucine degradation, and tyrosine metabolism, were significantly differentially expressed (*p* < 0.05). This suggests that these two pathways are potentially major factors in the regulation of tolerance to salt stress. In conclusion, the modulation of amino acid metabolism in *M. sativa* and *M. truncatula* can significantly increase their resistance to salt stress

### 3.3. Roles of Amino Acids in M. sativa and M. truncatula Under Salt Stress

In this study, the contents of three amino acids, Leu, Ile, and Tyr, were determined to investigate their potential functions and roles under salt stress. Their contents increased significantly in *M. sativa* and *M. truncatula* seedlings after salt stress treatment. Moreover, the amino acid content in plant roots was lower than that in the leaves, which may be owing to the transport of amino acids from the roots to the leaves during plant growth.

Many studies have shown that the accumulation of amino acids in the plant cytoplasm can increase the cellular water potential to some extent, thus reducing the harmful effects of osmotic stress on plants [50]. Yang et al. [51] found that various amino acids, such as Tyr, Leu, and Ile, accumulated in the leaves of soybean seedlings after salt stress. Huang et al. [52], when assessing the salt tolerance of *Arabidopsis thaliana* seedlings, found that the accumulation of branched-chain amino acids (Leu, Ile, and Val) increased, and the accumulation of certain amino acids even exceeded that of Pro. Wang et al. [53] analyzed the transcriptome and metabolome of *Zygomyrrhiza glabra* and observed a significant increase in Leu and Ile accumulation under salt stress and demonstrated that their respective metabolic pathways could be involved in the plant’s enhanced salt tolerance. These results were consistent with those in this study, indicating that amino acid content increases significantly under salt stress.

This study assessed the effects of the exogenous application of three amino acids, Leu, Ile, and Tyr, on salt stress alleviation in *M. sativa* and *M. truncatula*. Leu and Ile are branched-chain amino acids tightly associated with carbohydrate and energy metabolism. Tyr is an aromatic α-amino acid that plays an important role in the metabolism of the plant photosynthetic system and can facilitate the transition from Photosystem I to Photosystem II in photosynthesis [54]. As legumes, *M. sativa* and *M. truncatula* interact with rhizobacteria in the soil to form rhizobial symbioses during their growth and development. It was demonstrated that under salt stress, the amino acid content in the nodule sites of rhizobial symbionts increased significantly, including tyrosine among AAAs (aromatic amino acids) and leucine and isoleucine among BCAAs (branched-chain amino acids) [55,56]. Moreover, it has been shown in peas (*Pisum sativum* L.) that the BCAAs stimulate the formation of nodule sites, and their persistence is regulated by the host plant’s response to the growth environment [57]. BCAAs have also been shown to replace one of the respiratory pathways in plant cells and can provide energy to the plant under salt stress [58,59]. Bertrand et al. [60] found that in *M. sativa* under salt stress, BCAAs accumulated and provided the plant with energy to regulate ion transport and maintain osmotic balance, decreasing salt stress toxicity. BCAAs have also been shown to act as osmolytes in salt tolerance in addition to proline. In addition, the results showed that the biosynthesis of BCAAs plays an important role in the development of gametophytes and roots; thus, BCAAs help improve plants’ adaptation to salt stress [61]. Moreover, as a key factor in the production of secondary metabolites in plants, tyrosine plays an important role in promoting plant growth and development and regulating plants under abiotic stresses, including salt stress [62]. Therefore, in this study, we hypothesized that Leu and Ile, among the BCAAs, and tyrosine, among the AAAs, may be the critical factors for enhancing salt tolerance in *M. sativa* and *M. truncatula*

In this study, we demonstrated that three exogenous amino acids significantly reduced salt stress damage in *M. sativa* and *M. truncatula* seedlings compared to the control. Scarponi [63] found that exogenous application of Leu and Ile to soybeans could effectively enhance their salt tolerance. Exogenous Tyr stimulation accelerated the biosynthesis of phenolic secondary metabolites in meristems, ameliorating salt stress [9,64,65]. These findings are consistent with the results of this study. Our findings also revealed that the activities of SOD, CAT, and POD were reduced by the exogenous application of Leu to *M. sativa* treated with 50 mM NaCl. This indicates that Leu has a significant effect on *M. sativa* salt tolerance. Moreover, the exogenous application of Tyr in *M. truncatula* treated with 50 mM NaCl resulted in the lowest TBARS content and SOD and CAT activities. The results indicated that Tyr could be involved in the repair of the damage to the cell membrane and significantly alleviate the damage induced by salt stress in *M. truncatula*.

In summary, salt stress significantly inhibited the growth of *M. sativa* and *M. truncatula*. The transcriptome and proteomic analyses revealed that amino acid metabolic pathways were significantly differentially expressed in *M. sativa* and *M. truncatula* seedlings under salt stress. The glutathione metabolic pathway may significantly contribute to detoxification responses by differentially expressed *GSTs*, thereby enhancing plant tolerance to salinity. *ALDHs* and *AGXTs* with ROS scavenging functions were significantly differentially expressed in the valine, leucine, and isoleucine metabolic pathways and could alleviate the damage under salt stress in *M. sativa* and *M. truncatula*. *TDCs* exhibited differential expression in tyrosine metabolism, which promoted the production of tyramine to enhance plants’ tolerance to salt. In addition, the exogenous application of three amino acids, Leu, Ile, and Tyr, significantly improved *M. sativa* and *M. truncatula* tolerance to salinity. Notably, Leu significantly enhanced the salt tolerance of *M. sativa*, while Tyr significantly increased the tolerance of *M. truncatula*.

## 4. Materials and Methods

### 4.1. Plant Growth Conditions and Stress Treatment

Two varieties, Chifeng F2 (*M. sativa Medicago satica* L.) and r108 (*M. truncatula Medicago truncatula* L.), were used as the experimental plant materials. We obtained the *M. sativa* varieties from the Chifeng region of Inner Mongolia through self-propagation, while the *M. truncatula* varieties were obtained from the Yangzhou University (Yangzhou, China) experimental genotype collection (32.38° N, 119.42° E).

In this study, *M. sativa* and *M. truncatula* seeds were treated with 0 mM (control) and 50 mM NaCl. The treatments were labeled and divided into four groups based on the species and salt treatments, namely S0 (*M. sativa* control group), T0 (*M. truncatula* control group), S50 (*M. sativa* under 50 mM NaCl treatment), and T50 (*M. truncatula* under 50 mM NaCl treatment), with three replicates for each treatment group. We ensured that the seeds germinated at the designated concentrations of NaCl applied during the seed germination test by placing a thin sponge and a layer of filter paper inside the germination box. The boxes were placed in an artificial incubator that maintained a day/night temperature of 25 °C/20 °C. Each treatment contained 50 seeds that were irrigated with distilled water. After germination of the seeds, seedlings were transferred to a Hoagland nutrient solution with a NaCl concentration of 50 mM, which was replenished every three days to ensure that the NaCl concentration was maintained at 50 mM. Seedlings with good morphology and intact roots and leaves were selected from all the seeds that germinated as samples for the subsequent measurements of various indices, and 3 g of whole seedlings was selected from each treatment group and placed at −80 °C as a reserve sample. The number of seeds germinated was counted daily for the first 7 days, and the growth index, germination rate, and germination potential were measured after 7 days. The calculation formulas were as follows:

Growth index (%) = (N_1_ + N_2_/2 + N_3_/3 + N_4_/4 + N_5_/5 + N_6_/6 + N_7_/7) × 100%

Germination rate (%) = N_7_/(50 − H) × 100%

Germination potential = N_4_/(50 − H) × 100%

where N_1_: total number of seeds germinated on the first day. N_2_: total number of seeds germinated on the second day. N_3_: total number of seeds germinated on the third day. N_4_: total number of seeds germinated on the fourth day. N_5_: total number of seeds germinated on the fifth day. N_6_: total number of seeds germinated on the sixth day. N_7_: total number of seeds germinated on the seventh day. H: number of hard seeds (seeds in which the seed coat is impermeable to water and cannot swell to germinate, so remain at their original size).

Note: Germination rate represents the percentage of all seeds germinating after 7 days, while germination potential represents the percentage of all seeds germinating halfway through the test period. A higher germination rate indicates more viable seeds and higher seedling emergence after sowing. On the other hand, a high germination potential indicates a good rate and uniformity of germination and a high viability of the seeds.

### 4.2. Transcriptome Sequencing and Data Analysis

In this study, each treatment group was composed of three independent biological replicates. The RNA integrity was assessed using the RNA Nano 6000 Assay Kit on the Bioanalyzer 2100 system (Agilent Technologies, Santa Clara, CA, USA). The total RNA was used as the input material for the RNA sample preparations. Briefly, the mRNA was purified from the total RNA using poly-T oligo-attached magnetic beads. The mRNA was fragmented using divalent cations under elevated temperature in the first-strand synthesis reaction buffer (5×). First-strand cDNA was synthesized using random hexamer primers and M-MuLV reverse transcriptase (RNase H-). Second-strand cDNA was subsequently synthesized using DNA polymerase I and RNase H. Exonuclease/polymerase activities converted the remaining overhangs into blunt ends. After adenylation of the 3′ ends of DNA fragments, adaptors with a hairpin loop structure were ligated to prepare for hybridization. To select cDNA fragments of preferentially 370~420 bp in length, the library fragments were purified with the AMPure XP system (Beckman Coulter, Brea, CA, USA). PCR was performed with Phusion high-fidelity DNA polymerase, Universal PCR primers, and Index (X) primer. Finally, the PCR products were purified (AMPure XP system), and the library quality was assessed on the Agilent Bioanalyzer 2100 system. The index-coded samples were clustered on a cBot Cluster Generation System using the TruSeq PE Cluster Kit v3-cBot-HS (Illumina, San Diego, CA, USA) according to the manufacturer’s instructions. After cluster generation, the library preparations were sequenced on an Illumina NovaSeq platform, and 150 bp paired-end reads were generated.

The sequencing platform utilized in this study was the Illumina NovaSeq 6000 (Illumina, San Diego, CA, USA) provided by NovoZero Beijing (Beijing, China). The reference genome and gene model annotation file can be found at ftp://ftp.ensemblgenomes.org/pub/plants/release-55 (accessed on 14 July 2021). The reference genome was indexed using HISAT2 (HISAT2-v2.0.5) [66], and paired-end clean reads were aligned to the reference genome using HISAT2. featureCounts (1.5.0-p3) was used to calculate the number of reads mapped to each gene. The FPKM for each gene was then calculated based on the gene length, and the number of reads mapped to that gene was calculated. Genes with an adjusted *p*-value ≤ 0.05 and fold change ≥ 2 were determined as DEGs by DESeq2 (DESeq 2-1.20.0) [67]. A GSEA analysis was performed on the GO and Kyoto Encyclopedia of Genes and Genomes (KEGG) datasets for this species separately using the native version of the GSEA analysis tool [68] (gsea-3.0, http://www.broadinstitute.org/gsea/index.jsp (accessed on 14 July 2021)).

### 4.3. Proteome Profiling and Statistical Analysis

The total proteins were extracted using the TCA/acetone extraction protocol. Whole seedling samples (0.1 g) were ground individually in liquid nitrogen and lysed with SDT lysis buffer (4% SDS, 100 mM DTT, and 10 mM TEAB), followed by 5 min of ultrasonication on ice with three replicates for each treatment group. After incubation at 95 °C for 8 min, the lysate was centrifuged at 12,000× *g* for 15 min at 4 °C. The supernatant was reduced with 10 mM DTT for 1 h at 56 °C and subsequently alkylated with sufficient iodoacetamide for 1 h at room temperature in the dark. The samples were then thoroughly mixed with four volumes of precooled acetone by vortexing and incubating at −20 °C for at least 2 h. The samples were then centrifuged at 12,000× *g* for 15 min at 4 °C, and the precipitate was collected. After washing with 1 mL cold acetone, the pellet was dissolved by dissolution buffer (8 M urea and 100 mM TEAB, pH 8.5).

Each protein sample was taken, and the volume was brought to 100 μL with a DB dissolution buffer. Amounts of 0.002 g trypsin and 100 mM TEAB buffer were added, and the samples were mixed and digested at 37 °C for 4 h. Subsequently, 0.002 g trypsin and 0.1 g CaCl_2_ were added, and the samples were digested overnight. Formic acid was added to the digested samples, and the pH was adjusted to below 3. The samples were centrifuged at 12,000× *g* for 5 min at room temperature. The supernatant was slowly loaded to the C18 desalting column, washed three times with washing buffer (0.1% formic acid and 3% acetonitrile), and then eluted with elution buffer (0.1% formic acid and 70% acetonitrile). The eluents of each sample were collected and lyophilized. A volume of 100 μL of 0.1 M TEAB buffer was added to reconstitute the sample, and 41 μL of acetonitrile-dissolved TMT labeling reagent (0.5 mg TMT dry powder redissolved in 50 μL acetonitrile) was added. The samples were then mixed with shaking for 2 h at room temperature. The reaction was stopped by adding 9 μL 8% ammonia. All the labeled samples were mixed with an equal volume, desalted, and lyophilized.

The total proteins obtained were subjected to TMT proteomics analysis using the database 929452-merge.fasta (*M. sativa*: https://figshare.com/articles/dataset/Medicago_sativa_genome_and_annotation_files/12623960, *M. truncatula*: https://www.ncbi.nlm.nih.gov/datasets/genome/GCA_002024945.1/ (accessed on 14 July 2021)) [69,70] and the library search software Proteome Discoverer 2.4 (mass spectrometer model: Q Exactive^TM^HF-X (Thermo Fisher Scientific, Waltham, MA, USA)). To improve the quality and accuracy of the analytical results and reduce the false positive rate, the search results were further filtered with the Proteome Discoverer software: peptides with a peptide spectrum match (PSM) confidence score of 99% or more were considered plausible PSMs, and proteins containing at least one unique peptide were considered plausible proteins. Only plausible spectral peptides and proteins were retained. FDR data validation was performed to remove peptides and proteins with FDR greater than 1%.

A Gene Ontology (GO) functional analysis was conducted using the InterProScan [71] program (https://www.ebi.ac.uk/interpro/result/InterProScan/ (accessed on 14 July 2021)) against non-redundant protein databases, including Pfam, PRINTS, ProDom, SMART, ProSite, and PANTHER, and the databases of KEGG were used to analyze the protein family and pathway. The DEPs were used for the Volcano plot, cluster heat map, and GO and KEGG enrichment analyses. Putative protein–protein interactions were predicted using the STRING-db server [72] (http://string-db.org/ (accessed on 14 July 2021)).

### 4.4. Determination of Amino Acids

The leaves and roots of *M. sativa* and *M. truncatula* were separated from the plants for amino acid determinations. Three biological replicates were assessed from each treatment group. The experimental method was as follows: approximately 50 mg of the sample was weighed; one steel bead and 10 ceramic beads were added, and the samples were homogenized for 1 min. A volume of 1 mL of an aqueous solution of 50% methanol was added to the samples, which were then extracted by vortexing and shaken for 1 h. The centrifuge tubes with the samples were centrifuged for 10 min at 4 °C and 12,000× *g*. A volume of 10 μL of the supernatant was collected and transferred to a 1.5 mL centrifuge tube. A volume of 10 μL of ultrapure water, 5 μL of internal standard, and 40 μL of isopropanol (0.1% formic acid) were added, and the samples were vortexed for 2 min. After that, the tubes were centrifuged for 10 min at 4 °C and 12,000× *g*. A volume of 10 μL of the supernatant was collected and placed in a 1.5 mL centrifuge tube, and 70 μL of boric acid buffer salt and 20 μL of AccQ Tag derivatizations reagent (Kairos amino acid kit, Waters, Milford, MA, USA) were added. The tubes were immediately shaken for 10 s. After 1 min, the excess derivatization agent was hydrolyzed, and the derivatization reaction was finished. The centrifuge tubes were heated at 55 °C for 10 min. A volume of 400 μL of water was then added to dilute the sample for measurement.

The gradient dilution method was used to prepare the standards and sequentially prepared solutions of each amino acid at 400, 200, 100, 40, 20, 10, 4, 2, and 1 μmol/L concentrations. Before the measurements, we added and mixed an equal volume of a stable isotope (tyrosine: 13C9, 99%, 15N, 99%; leucine: 13C6, 99%, 15N, 99%; and isoleucine: 13C6, 99%, 15N, 99%) internal standard. The samples were purified by chromatography with a Waters ACQUITY UPLC I-CLASS ultra-performance liquid chromatography system, followed by a mass spectrometry analysis using the Waters XEVO TQ-S tandem quadrupole mass spectrometry system. The positive ion source voltage was 3 kV, while the cone-well voltage was 10 V. The desolventization temperature was 500 °C; the desolventization gas flow rate was 1000 L/h, and the cone-well gas flow rate was 10 L/h. The peak areas were calculated using MassLynx software (version 4.3) for quantitative data processing. The retention time was allowed to have an error of 15 s. Quantitative results were obtained using a standard curve.

### 4.5. Integrated Transcriptome and Proteome Analysis

A correlation analysis (R-3.4.3) was also performed for the differential ploidy of genes (proteins) identified jointly by the transcriptome and proteome in the two species of *Medicago*. Finally, GO and KEGG enrichment analyses (R-3.4.4, gsea-3.0) were performed for the collected data.

### 4.6. Assays of the Antioxidant Enzymes and Other Physiological and Biochemical Indices

Superoxide dismutase (SOD) was assayed using the nitrogen blue tetrazolium method [73]. Peroxidase (POD) was assayed using the guaiacol colorimetric method [74]. Catalase (CAT) was assayed using the ammonium molybdate colorimetric method [75]. The tiobarbituric acid-reactive substances (TBARS) content was determined using thiobarbituric acid [76]. All the indicators of enzyme activity were measured individually using Suzhou Keming Biotechnology kits (Suzhou, China). The concentration of Na^+^ and K^+^ was delivered to the Beijing Novogene Company (Beijing, China) for determination.

### 4.7. Statistical Analysis

All data from this study were compiled using Microsoft Excel 2007 (Microsoft, Redmond, WA, USA), and the statistical analyses were performed using SPSS 18.0 (Chicago, IL, USA). Since there are two independent variables in this test, a two-way analysis of variance (ANOVA) and *t*-test were more appropriate to determine statistically significant differences among the treatment groups, with *p* < 0.05 as the criterion for significance.

## 5. Conclusions

This study revealed that the detoxification reactions catalyzed by *GSTs* binding to glutathione (*GSH*) scavenge excess reactive oxygen species from the cells via *ALDHs* and *AGXTs*. At the same time, the biosynthesis of tyramine by the *TDCs* jointly contributes to enhancing salt tolerance in *M. sativa* (*Medicago sativa* L.) and *M. truncatula* (*Medicago truncatula* L.). Leu exerted a more pronounced influence on mitigating the toxicity of salt stress during the germination of *M. sativa* seeds. Conversely, Tyr was more effective at mitigating the damage caused by salt stress during the germination of *M. truncatula* seeds. This study provides a comprehensive analysis of the salt tolerance mechanisms in *M. sativa* and *M. truncatula*, which is highly significant in guiding the future breeding of salt-tolerant alfalfa varieties.

## 6. Future Perspectives

Future research should prioritize the integration of metabolomics data to elucidate the mechanisms of amino acid metabolism related to salt resistance in *M. sativa* (*Medicago sativa* L.) and *M. truncatula* (*Medicago truncatula* L.). Additionally, it should focus on developing machine learning models to integrate transcriptomic, proteomic, and metabolomic data to predict key nodes of amino acid metabolism regulation. For example, the ZmbZIP transcription factor in maize was demonstrated to activate the *GSH1* gene, facilitating *GSH* synthesis and enhancing ROS scavenging and detoxification [77].

Secondly, molecular marker-assisted breeding research should be conducted, based on the joint transcriptome–proteome analysis of this study, to identify and utilize SNP markers related to salt tolerance (e.g., the *GST* gene family of variant sites mentioned in this study) for rapid selection of salt-tolerant varieties in *M. sativa* and *M. truncatula*.

Further, as *M. sativa* and *M. truncatula* belong to the legume family, it is important to study how inter-root microorganisms (e.g., salt-tolerant rhizobacteria) modulate amino acid metabolism-related gene expression in plants through the secretion of metabolites and to develop synergistic microorganism–plant formulations to enhance plant salt-tolerance.

Finally, high-throughput phenomics and multi-omics data should be combined to establish a salt tolerance prediction model, conduct salt adaptation trials, and observe the phenotypes and yields of actual salt-tolerant varieties in saline soils in the field.

## 7. Limitations

Firstly, there were frequent inconsistencies between mRNA expression levels and corresponding protein abundance in this study, potentially due to post-translational modifications, protein degradation, and differences in translational efficiency. Consequently, reliance solely on transcriptome data may result in an overestimation or underestimation of the actual activity of key metabolic enzymes.

Secondly, this study conducted sampling and subsequent experimentation only 7 days after seed germination, potentially overlooking critical nodes of amino acid metabolism in plants acclimated to long-term salt stress.

Further, *M. sativa* (*Medicago sativa* L.) is a tetraploid, and there is the possibility of redundancy of gene functions of members of related gene families involved in amino acid metabolism. These issues may hinder the phenotyping of a single gene knockout and affect its functional validation.

Meanwhile, the transcriptomics and proteomics data could not reflect the dynamic changes in metabolic flow, limiting the understanding of metabolic homeostatic regulation.

Finally, plants under salt stress frequently endure additional compound stresses such as drought and ionic toxicity. Therefore, it is difficult to simulate the complex environment in the field under a single stress condition in the laboratory, which leads to some limitations on the applicability of the data studied in the field.

## Figures and Tables

**Table 1:** Root length (cm), seedling length (cm), SOD (u/mg prot), CAT (μmol/min/mg prot), POD (u/mg prot) activities, and TBARS (mmol/mg prot) content of *M. sativa* and *M. truncatula* seedlings grown for 7 days after the exogenous application of Leu.

	*M. sativa* (*Medicago sativa* L.)				*M. truncatula* (*Medicago truncatula* L.)			
Treatments	Control	NaCl	Leu	Leu + NaCl	Control	NaCl	Leu	Leu + NaCl
Root	2.10 c ± 0.03	1.16 f ± 0.01	2.51 a ± 0.01	1.71 e ± 0.02	1.94 d ± 0.02	0.84 g ± 0.02	2.39 b ± 0.00	1.22 f ± 0.03
Seedling	3.7 3c ± 0.05	2.42 e ± 0.02	4.10 b ± 0.01	2.79 d ± 0.01	3.87 d ± 0.01	2.31 d ± 0.01	4.41 a ± 0.04	2.79 d ± 0.01
SOD	57.36 c ± 1.51	72.65 a ± 0.64	45.19 d ± 0.41	65.53 b ± 0.76	52.81 b ± 0.76	63.66 b ± 0.76	40.45 e ± 0.73	57.53 e ± 1.12
CAT	7.29 e ± 0.16	28.73 a ± 0.29	4.90 f ± 0.08	10.66 d ± 0.18	15.83 d ± 0.18	31.65 d ± 0.18	12.92 c ± 0.06	20.46 b ± 0.15
POD	0.63 bc ± 0.01	0.82 a ± 0.02	0.41 e ± 0.02	0.63 bc ± 0.01	0.66 bc ± 0.01	0.79 bc ± 0.01	0.53 d ± 0.01	0.62 c ± 0.01
TBARS	0.41 c ± 0.03	0.52 b ± 0.04	0.22 d ± 0.01	0.52 b ± 0.03	0.42 b ± 0.03	0.72 b ± 0.03	0.23 d ± 0.01	0.71 a ± 0.01

Note: Data are presented as the mean ± SE of three replicates (*n* = 3). The units of measurement are expressed as mmol/g, where ’g’ denotes the fresh weight of the sample. Means followed by different letters in the same row are significantly different (*p* < 0.05). CAT, catalase; Leu, leucine; TBARS, tiobarbituric acid-reactive substances; POD, peroxidase; SOD, superoxide dismutase.

**Table 2:** Root length (cm), seedling length (cm), SOD (u/mg prot), CAT (μmol/min/mg prot), POD (u/mg prot) activities, and TBARS (mmol/mg prot) content of *M. sativa* and *M. truncatula* seedlings grown for 7 days after the exogenous application of Ile.

	*M. sativa* (*Medicago sativa* L.)				*M. truncatula* (*Medicago truncatula* L.)			
Treatments	Control	NaCl	Ile	Ile + NaCl	Control	NaCl	Ile	Ile + NaCl
Root	2.10 d ± 0.03	1.16 f ± 0.01	2.41 a ± 0.01	1.58 e ± 0.02	1.94 d ± 0.02	0.84 d ± 0.02	2.29 c ± 0.00	1.16 f ± 0.03
Seedling	3.73 c ± 0.05	2.42 f ± 0.02	3.99 b ± 0.01	2.69 d ± 0.02	3.87 d ± 0.01	2.31 d ± 0.01	4.39 a ± 0.01	2.62 e ± 0.03
SOD	57.36 d ± 1.51	72.65 a ± 0.64	49.22 e ± 0.75	66.21 b ± 0.97	52.81 b ± 0.76	63.66 b ± 0.76	41.17 f ± 0.83	58.79 cd ± 0.58
CAT	7.29 e ± 0.16	28.73 a ± 0.29	5.31 f ± 0.03	21.19 c ± 0.54	15.83 d ± 0.18	31.65 d ± 0.18	12.90 d ± 0.09	24.65 b ± 0.45
POD	0.63 e ± 0.01	0.82 a ± 0.02	0.46 g ± 0.01	0.68 c ± 0.01	0.66 bc ± 0.01	0.79 bc ± 0.01	0.54 f ± 0.02	0.64 de ± 0.01
TBARS	0.41 c ± 0.03	0.52 b ± 0.04	0.23 d ± 0.01	0.54 b ± 0.02	0.42 b ± 0.03	0.72 b ± 0.03	0.24 d ± 0.01	0.68 a ± 0.02

Data are presented as the mean ± standard error of three replicates (*n* = 3). The units of measurement are expressed as mmol/g, where ’g’ denotes the fresh weight of the sample. Means followed by different letters in the same row are significantly different (*p* < 0.05). CAT, catalase; Ile, isoleucine; TBARS, tiobarbituric acid-reactive substances; POD, peroxidase; SOD, superoxide dismutase.

**Table 3:** Root length (cm), seedling length (cm), SOD (u/mg prot), CAT (μmol/min/mg prot), POD (u/mg prot) activities, and TBARS (mmol/mg prot) content of *M. sativa* and *M. truncatula* seedlings grown for 7 days after the exogenous application of Tyr.

	*M. sativa* (*Medicago sativa* L.)				*M. truncatula* (*Medicago truncatula* L.)			
Treatments	Control	NaCl	Tyr	Tyr + NaCl	Control	NaCl	Tyr	Tyr + NaCl
Root	2.10 c ± 0.03	1.16 f ± 0.01	2.35 b ± 0.03	1.52 d ± 0.01	1.94 d ± 0.02	0.84 d ± 0.02	2.42 a ± 0.01	1.29 e ± 0.01
Seedling	3.73 c ± 0.05	2.42 f ± 0.02	3.96 b ± 0.06	2.61 e ± 0.02	3.87 d ± 0.01	2.31 d ± 0.01	4.39 a ± 0.02	2.88 d ± 0.03
SOD	57.36 d ± 1.51	72.65 a ± 0.64	53.76 e ± 1.26	62.45 c ± 1.23	52.81 b ± 0.76	63.66 b ± 0.76	34.70 f ± 0.67	52.45 e ± 1.08
CAT	7.29 f ± 0.16	28.73 a ± 0.29	5.77 g ± 0.05	23.83 b ± 0.29	15.83 d ± 0.18	31.65 d ± 0.18	11.53 e ± 0.04	19.05 d ± 0.50
POD	0.63 e ± 0.01	0.82 a ± 0.02	0.47 g ± 0.01	0.75 c ± 0.01	0.66 bc ± 0.01	0.79 bc ± 0.01	0.50 f ± 0.01	0.65 de ± 0.01
TBARS	0.41 d ± 0.03	0.52 c ± 0.04	0.24 e ± 0.01	0.53 c ± 0.01	0.42 b ± 0.03	0.72 b ± 0.03	0.22 e ± 0.01	0.65 b ± 0.01

Data are presented as the mean ± standard error of three replicates (*n* = 3). The units of measurement are expressed as mmol/g, where ’g’ denotes the fresh weight of the sample. Means followed by different letters in the same row are significantly different (*p* < 0.05). CAT, catalase; TBARS, tiobarbituric acid-reactive substances; POD, peroxidase; SOD, superoxide dismutase; Tyr, tyrosine.

## Data Availability

The transcriptome datasets generated and analyzed during the current study are available in the NCBI repository, the accession number is PRJNA1050396. The proteome datasets generated and analyzed during the current study are available in the iProX repository, and the accession number is PXD047690. The datasets generated for this study are available on request to the corresponding author.

## Supplementary Materials

The following supporting information can be downloaded at: https://www.mdpi.com/article/10.3390/plants14060929/s1, Figure S1. Reproducibility of transcriptomics based on the correlations among biological replicates. Figure S2. Principal component analysis of the transcriptomics dataset. Figure S3. Heatmap of the transcriptomics dataset. Figure S4. GSEA analysis of the transcriptomics. Figure S5. Principal component analysis of the proteomics dataset. Figure S6. Heatmap of the proteomics dataset. Table S1. Germination and growth of *M. sativa* and *M. truncatula* seedlings. Table S2. Comparison of DEGs involved in amino acid metabolic pathways identified in the transcriptomic data by treatment. Table S3. Specific information on DEGs involved in amino acid metabolism. Table S4. Overview of proteins identified from the proteome sequencing data. Table S5. DEPs involved in amino acid metabolic pathways were identified in the comparative proteomics dataset for each treatment comparison group. Table S6 Specific information on DEPs involved in amino acid metabolism. Table S7. Classification of the KEGG pathways identified in the combined transcriptomic and proteomic analysis.

## Abbreviations

CAT	Catalase
DEG	Differentially expressed genes
DEP	Differentially expressed proteins
DMSO	Dimethyl sulfoxide
DTT	Dithiothreitol
FDR	False discovery rate
FPKM	Fragments per kilobase of exon model per million mapped fragments
Glu	Glutamic acid
GSEA	Gene set enrichment analysis
GO	Gene Ontology
Ile	Isoleucine
KEGG	Kyoto encyclopedia of genes and genomes
Leu	Leucine
Na^+^	Sodium ions
NES	Normalised enrichment score
POD	Peroxidase
PSM	Peptide spectrum match
SOD	Superoxide dismutase
TBARS	tiobarbituric acid-reactive substances
TCA	tricarboxylic acid
TEAB	Tetraethylammonium bromide
TMT	Tandem mass tag
TOR	target of rapamycin
Tyr	Tyrosine
UV	Ultraviolet
