# Supplementary figures and images for: The Involvement of Amino Acid Metabolism in the Mechanisms of Salt Tolerance Adaptation in Medicago sativa and Medicago truncatula
Source: Plants (Basel). 2025 Mar 15;14(6):929. doi: 10.3390/plants14060929 (PMC11945280; doi:10.3390/plants14060929)

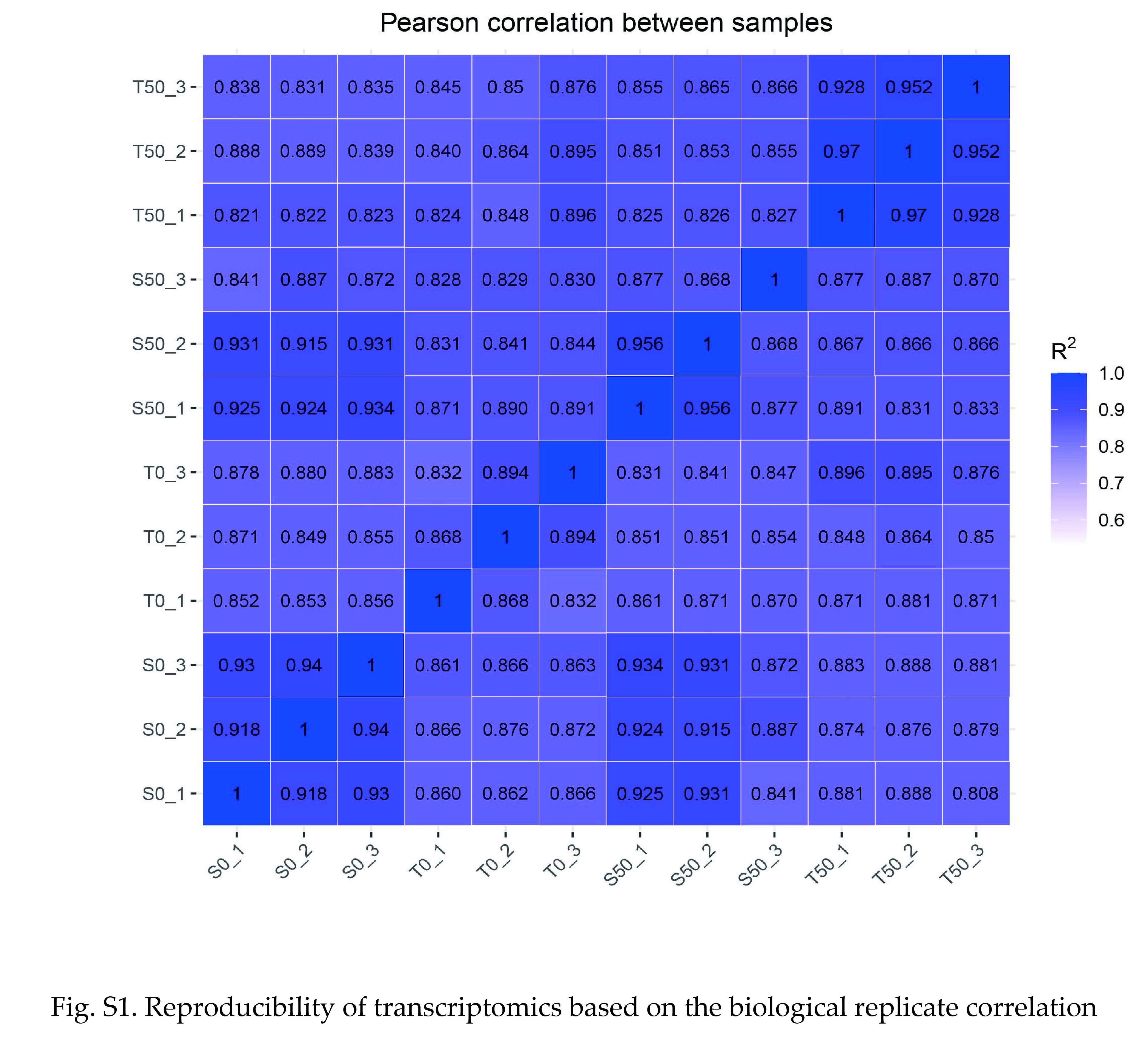

Supplement: Supplementary file 1 [file plants-14-00929-s001.zip › plants-3527690-Figure S1.jpg]

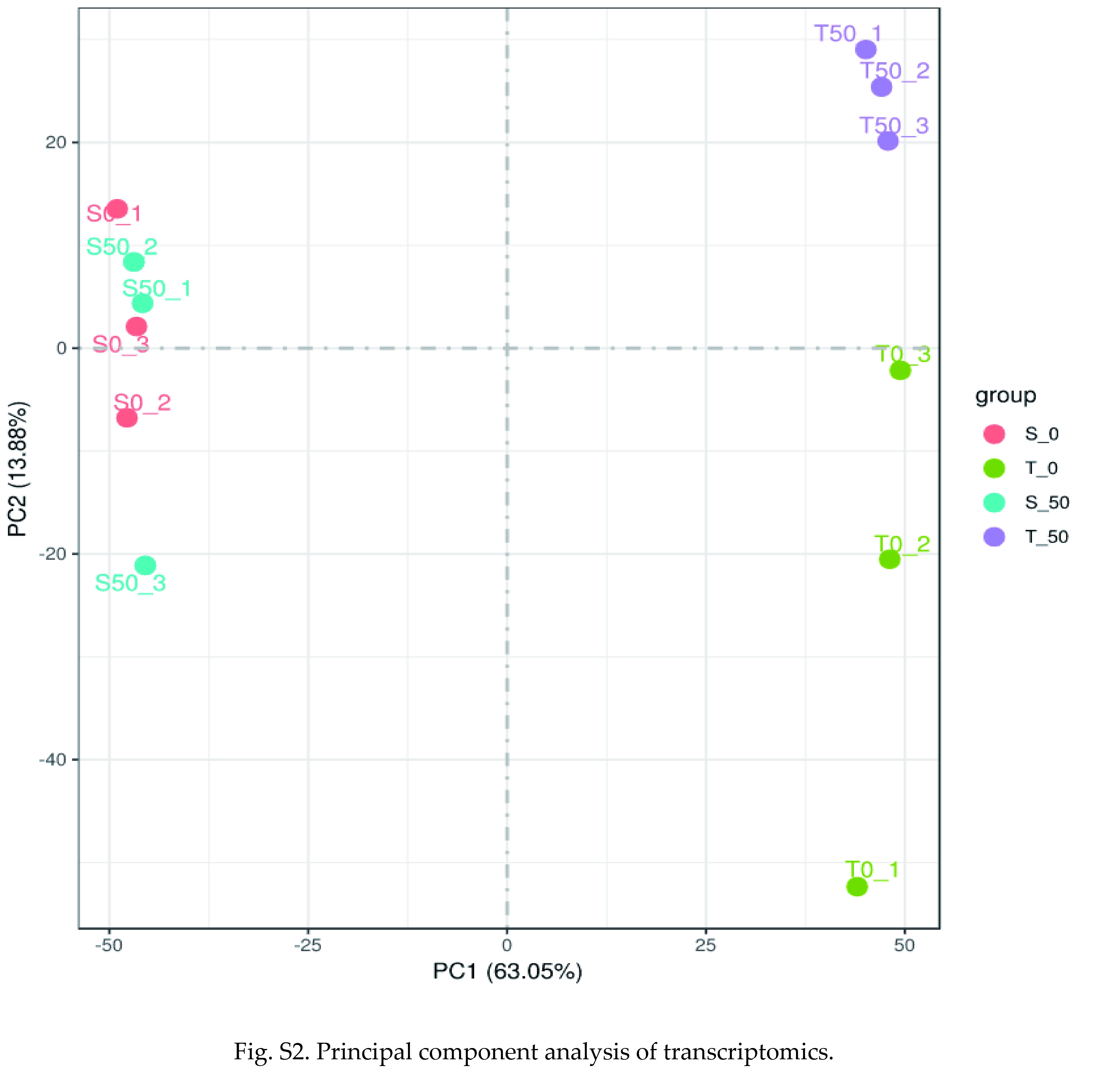

Supplement: Supplementary file 1 [file plants-14-00929-s001.zip › plants-3527690-Figure S2.jpg]

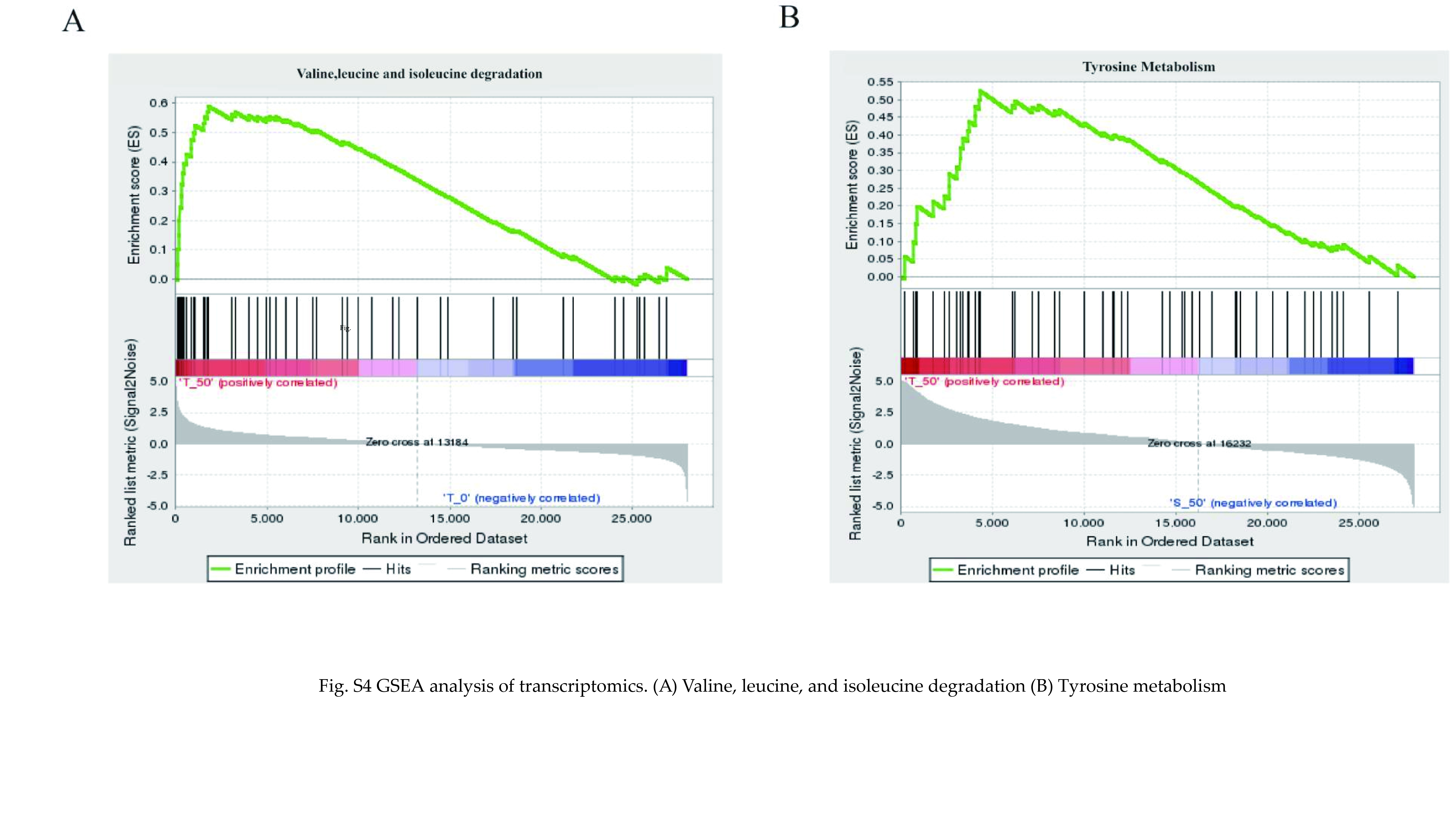

Supplement: Supplementary file 1 [file plants-14-00929-s001.zip › plants-3527690-Figure S4.jpg]

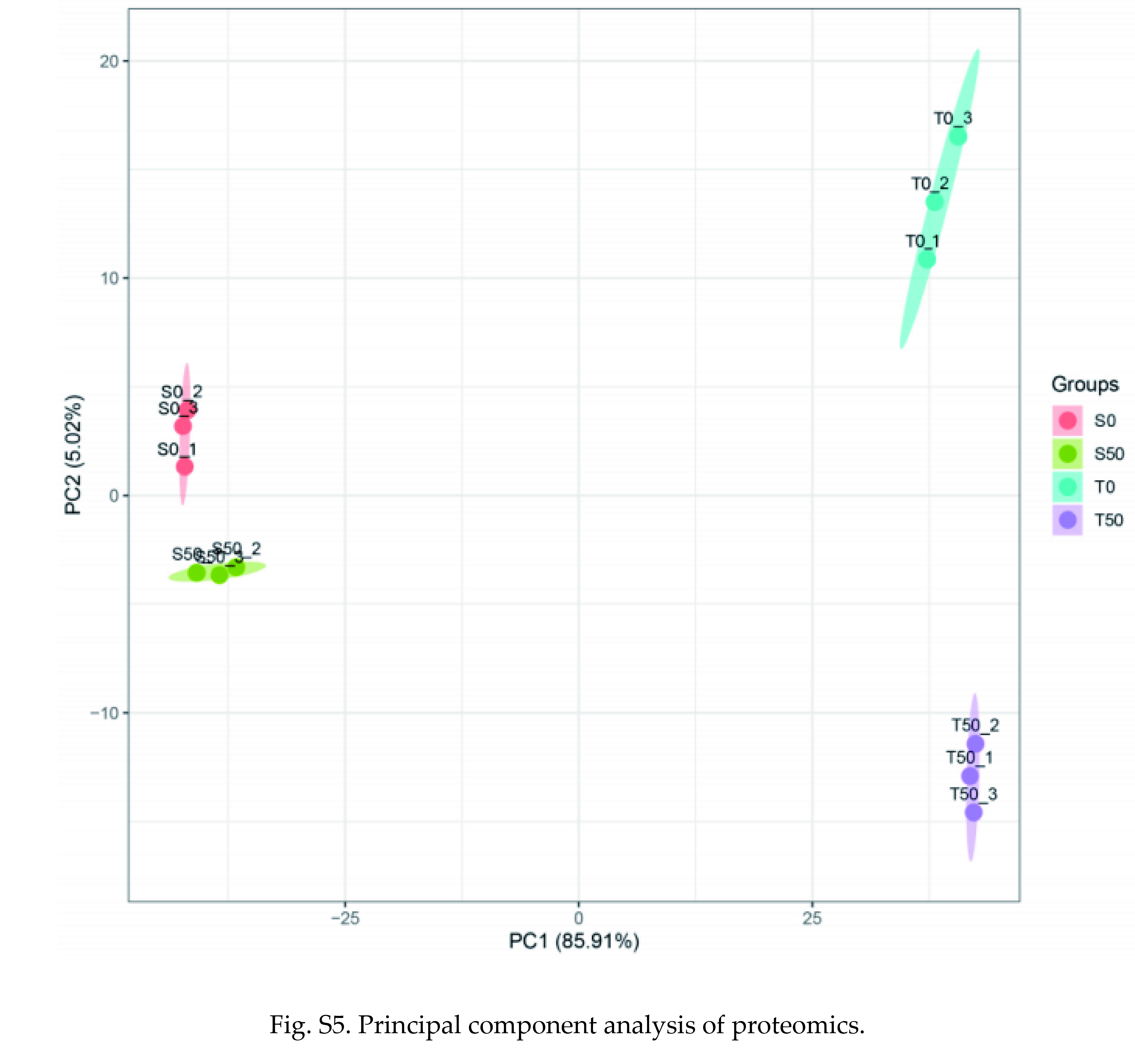

Supplement: Supplementary file 1 [file plants-14-00929-s001.zip › plants-3527690-Figure S5.jpg]

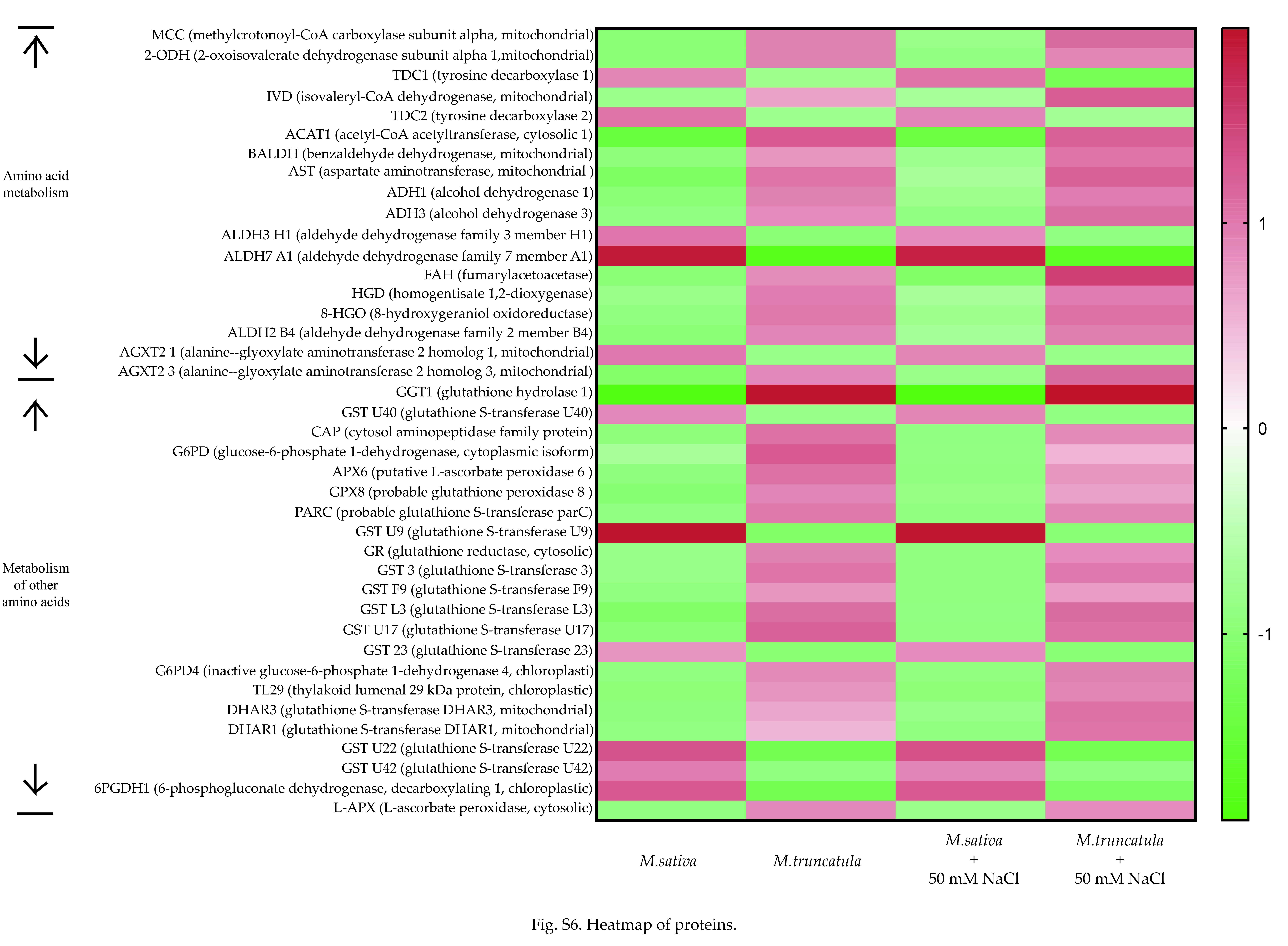

Supplement: Supplementary file 1 [file plants-14-00929-s001.zip › plants-3527690-Figure S6.jpg]
